# Delayed SARS-COV-2 clearance in infected persons in Ghana

**DOI:** 10.4314/gmj.v57i2.3

**Published:** 2023-06

**Authors:** Mildred A. Adusei-Poku, James O. Aboagye, Joseph H. K. Bonney, John K. Odoom, Evangeline Obodai, Helena Lamptey, Ivy A. Asante, Seth Agyemang, Joseph Oliver-Commey, George B. Kyei, William K. Ampofo, Evelyn Y. Bonney

**Affiliations:** 1 Department of Medical Microbiology, University of Ghana Medical School. College of Health Sciences, University of Ghana, Accra, Ghana; 2 Virology Department. Noguchi Memorial Institute for Medical Research, College of Health Sciences, University of Ghana. Off Akilagpa Sawyerr Road, Legon, Accra, Ghana; 3 Immunology Department. Noguchi Memorial Institute for Medical Research, College of Health Sciences, University of Ghana. Off Akilagpa Sawyerr Road, Legon, Accra, Ghana; 4 Korle Bu Teaching Hospital, Korle Bu, Accra, Ghana; 5 LEKMA Hospital, Agbleza Manet Road, Teshie, Accra, Ghana; 6 Department of Medicine, Washington University School of Medicine, 660 S. Euclid Avenue, St. Louis, MO, USA

**Keywords:** Virus clearance, SARS-CoV-2, RT-PCR, isolation, sputum

## Abstract

**Objective:**

This study aimed to determine the duration of SARS-CoV-2 clearance in persons in Ghana. The research question was whether the duration of virus clearance in Ghana matched the 14 days recommended by the World Health Organization (WHO); this had direct implications for transmission, which was key in managing the COVID-19 pandemic.

**Design:**

This was a retrospective analytical study.

**Setting:**

All facilities that submitted clinical specimens to Noguchi Memorial Institute for Medical Research (NMIMR) for SARS-CoV-2 diagnosis between March to June 2020 were included in the study.

**Interventions:**

Samples from 480 persons who tested positive for SARS-CoV-2 by RT-PCR from March to June 2020 at NMIMR and submitted at least two follow-up samples were retrospectively analysed. Individuals with two consecutive negative RT-PCR retesting results were considered to have cleared SARS-CoV-2.

**Results:**

The median time from the initial positive test to virus clearance was 20 days (IQR: 5-56 days). This was six days longer than the WHO-recommended 14 days, after which infected persons could be de-isolated. Sputum and nasopharyngeal swabs proved more sensitive for detecting viral RNA as the infection progressed. At a significance level of 0.05, age and sex did not seem to influence the time to SARS-CoV-2 clearance.

**Conclusions:**

The median time to SARS-CoV-2 clearance in this study was 20 days, suggesting that SARS-CoV-2 infected persons in Ghana take longer to clear the virus. This finding calls for further investigations into whether patients who remain PCR positive continue to be infectious and inform isolation practices in Ghana.

**Funding:**

The study was supported by the Ministry of Health/ Ghana Health Service through the provision of laboratory supplies, the US Naval Medical Research Unit #3, the World Health Organization, the Jack Ma Foundation and the Virology Department of Noguchi Memorial Institute for Medical Research, University of Ghana. Research projects within Noguchi Memorial Institute for Medical Research contributed reagents and laboratory consumables. However, the authors alone are responsible for the contents of this manuscript.

## Introduction

Coronavirus belongs to the family Coronaviridae, sub-family Coronavirinae.^1^ This family of viruses may cause symptoms such as myalgia, fever, breathing difficulty, and clinical syndromes such as the common cold and pneumonia.^2^ Coronaviruses are commonly found in animals, with very few species such as a severe acute respiratory virus (SARS-CoV) and the Middle East respiratory syndrome-related coronavirus (MERS-CoV) causing disease in humans.^3^

The World Health Organization (WHO) named the disease, caused by the novel coronavirus that affected the lower respiratory tract of patients with pneumonia in Wuhan, China, in December 2019, COVID-19.^4^ Severe acute respiratory syndrome coronavirus 2 (SARS-CoV-2), the causative agent for COVID-19 is now responsible for over 220 million infections and more than 4.7 million deaths worldwide as at September 20, 2021.^5^, ^6^ In Ghana there are over 125,000 confirmed cases of COVID-19 infections with 1,121 deaths.^7^ Most COVID-19-infected people are either asymptomatic or present with mild to moderate respiratory illness and recover without special treatment. However, older people and those with under-lying medical problems such as cardiovascular disease, diabetes, chronic respiratory disease, hypertension and cancer are more likely to develop serious illnesses.^8^ At the time of this data, there were no specific vaccines or treatments for COVID-19. However, many ongoing clinical trials were evaluating potential treatments.^9^

In January 2020, the WHO published its maiden guidelines for the clinical management of COVID-19, which included recommendations on when patients were no longer considered infectious and could be released from isolation.^10^ Here, patients were required to have been clinically recovered and have two negative RT-PCR test results taken sequentially in a 24-hour window. This recommendation was based largely on experience and previous knowledge of SARS and MERS.^10^

Ghana adopted and implemented the WHO's initial recommendation where patients were required to have two consecutive negative RT-PCR tests within a 24-hour window before they could be discharged from isolation.^10^ Later, some reports indicated that patients with mild COVID-19 infection shed the virus for approximately 7-12 days; this duration may be longer in severe cases though significant variations have been reported in both instances.^11^ A study by Wölfel *et al.* indicated that the virus could be isolated in culture from patient specimens (swabs or sputum) taken during the first week of symptoms but could not be isolated from specimens taken after the eighth day following symptoms though they were RT-PCR positive.^12^ Therefore, the virus might not be infectious after the eighth day following symptoms onset. He *et al.* corroborated this finding in their research, indicating that viral shedding may begin 5 to 6 days before the appearance of the first symptoms, and viral loads significantly decline after the onset of symptoms.^13^ These findings thus suggest that a positive RT-PCR result does not necessarily equate to infectiousness. Based on these and the fact that many countries, including Ghana, had logistical challenges, the WHO revised the criteria for releasing patients from isolation such that people in isolation could be de-isolated 14 days after the initial positive test without an exit RT-PCR test.^14^ Per this recommendation, symptomatic patients could also be discharged 14 days after the first positive test plus at least three days without symptoms.^14^

Although Ghana revised its discharge from isolation policy based on the updated WHO recommendations,^14^ it is worth noting that the data on which the new WHO recommendation was based came mainly from Asia, Europe and the USA, with none from Africa.^12^, ^13^ Since SARS-CoV-2 infection in Africa seems to have a different trajectory in most patients, with fewer deaths, it is important to determine the duration for viral clearance in African patients to help guide policies on the continent. Here, we present the results of virus clearance data for 480 people tested in Ghana during the first WHO criteria for de-isolation who were infected with SARS-CoV-2 and had at least two more RT-PCR tests after the initial positive test results.

## Methods

### Sample collection and laboratory testing

The Virology Department of the University of Ghana's Noguchi Memorial Institute for Medical Research (NMIMR) was the major testing centre for SARS-CoV-2 in Ghana. The samples reported here were submitted between March 12 and June 14, 2020, as part of Ghana's emergency response to the SARS-CoV-2 pandemic. Samples were accompanied by the Ghana Health Service case forms from which available demographic information was extracted. Sputum or oropharyngeal or nasopharyngeal swabs were collected from returning travellers, patients suspected of COVID-19, or close contacts of confirmed cases. The samples were submitted to NMIMR and taken through ribonucleic acid (RNA) extraction and real-time reverse transcription polymerase chain reaction (RT-PCR). The nucleocapsid (N), the open reading frame 1ab (ORF 1ab) and/or the envelope (E) genes of SARS-CoV-2 were amplified using gene-specific primers as designed by the manufacturer (Da An Gene, MiRXES, and TIBMOLBIOL). The RT-PCR results were interpreted as instructed by the kit manufacturers. Cycle threshold (Ct) values below 40 were considered positive for these kits. Internal controls were included as quality control for sample collection, RNA extraction and the PCR processes. In the database, 480 people with at least two test results after their initial positive test were analysed for this study. Out of these, those with two consecutive Negative retest results (419) were analysed for their various duration to virus clearance. Patients who tested positive were quarantined and required to submit subsequent samples for retesting after 14 days and at 7-day intervals (Days 14, 21, 28, etc.) until a negative test for SARS-CoV-2 was obtained.

By the World Health Organization's protocol at the time of this study, persons in quarantine needed two consecutive PCR negative results within 24 hours to be de-isolated. Clearance of SARS-CoV-2 in infected individuals was defined as having two consecutive negative PCR results after the initial positive result. These two consecutive Negative results were obtained at varying patient sampling times.

### Statistical analysis

Categorical variables were described as frequency and percentage, while continuous variables were expressed as the median and interquartile range (IQR). Statistical analyses were performed with SPSS version 22. The significance level was set at 0.05.

## Results

### Demographics and testing Outcomes

Ghana reported its first COVID-19 case on March 12 2020. As of June 14, 2020, our laboratory had confirmed 7,025 COVID-19 cases with follow-up samples from 1,073 patients. Four hundred and eighty (45%) out of the 1,073 had submitted at least two follow-up samples after testing positive.

Of the 480 individuals, 419 had two consecutive negative results (Table 1), while 61 still tested positive. Of the 419 patients, 54% (228) were 40 years or younger, and 55.6% were male (Table 1). Six patients out of 419 (1.4%) tested negative after submitting a new sample within a week (7 days), while 25% became RT-PCR negative two weeks after their first test (8-14 days). However, over 70% (308/419) tested negative after 14 days (Table 1). The median days to test negative for the patients in this study was 20 days (IQR, 5 - 56). Nearly 90% of the patients who became RT-PCR negative submitted only two samples following their first test.

**Table 1:** Characteristics of SARS-CoV-2 infected patients with two consecutive negative results

Variable	Number of patients n(%)	Testing negative within 14 days (%)	Median days (IQR)	OR (CI)	p Value	
**Gender**	Female	186 (44.4)	51 (27.4)	20 (5 - 56)	1.1 (0.7 - 1.7)	0.7007
	Male	233 (55.6)	60 (25.8)	19 (6 - 50)	1	
**Age (years)**	< 18	21 (5.0)	4 (19.0)	19 (11 - 47)	1	-
	18 - 40	207 (49.4)	53 (25.6)	20 (6 - 56)	1.5 (0.5 - 4.5)	0.5107
	> 40	118 (28.2)	33 (28.0)	20 (5 - 48)	1.7 (0.5 - 5.3)	0.3979
	Missing	73 (17.4)	21 (28.8)	19 (7 - 44)	1.7 (0.5 - 5.7)	0.3781
**Symptoms**	Asymptomatic	367 (87.6)	103 (28.1)	19 (5 - 47)	2.1 (1.0 - 4.7)	0.0572
	Symptomatic	52 (12.4)	8 (15.4)	21 (6 - 56)	1	-
**Number of follow-ups**	2 samples	378 (90.2)	109 (28.8)	19 (5 - 48)	5.7 (1.3 - 24.2)	0.0191
	3 samples	30 (7.2)	2 (6.7)	27 (13 - 50)	1	-
	4 samples	11 (2.6)	0 (0.0)	31 (21 - 56)	-	-
**Days to testing negative**	≤ 7 days	6 (1.4)	-	-	-	-
	8 - 14 days	105 (25.1)	-	-	-	-
	15 - 21 days	139 (33.2)	-	-	-	-
	> 21 days	169 (40.3)	-	-	-	-
	Total	419 (100.0)	111 (26.5)	20 (5 - 56)	-	-

### Factors associated with infected patients testing negative

To determine the risk associated with days to recovery, we assessed gender, age, presentation with symptoms, and the type of sample as a factor. There was no significant association between gender and age in testing negative within 14 days after being confirmed with SARS-CoV-2 infection (Table 1). Though not statistically significant, symptomatic patients required longer to test Negative than asymptomatic patients (Table1). To investigate what type of sample contributed to detecting the virus, oropharyngeal, nasopharyngeal, and sputum samples were collected from seventeen patients who still tested positive 21 days after the initial positive test. As shown in Table 2, eight patients were still SARS-CoV-2 positive.

**Table 2:** Repeat test results from oropharyngeal, nasopharyngeal and sputum specimens collected after 21 days from 17 patients who had previously tested positive for SARS-CoV-2 by RT-PCR

Patient	Specimen type and Results		
	Oropharyngeal	Nasopharyngeal	Sputum
**Patient 1**	Negative	Negative	Positive
**Patient 2**	Negative	Negative	Positive
**Patient 3**	Negative	Negative	Negative
**Patient 4**	Negative	Negative	Positive
**Patient 5**	Negative	Negative	Negative
**Patient 6**	Negative	Negative	Negative
**Patient 7**	Negative	Negative	Positive
**Patient 8**	Negative	Negative	Negative
**Patient 9**	Negative	Negative	Negative
**Patient 10**	Negative	Negative	Negative
**Patient 11**	Negative	Negative	Negative
**Patient 12**	Negative	Positive	Positive
**Patient 13**	Negative	Positive	Positive
**Patient 14**	Negative	Negative	Negative
**Patient 15**	Negative	Negative	Positive
**Patient 16**	Negative	Positive	Negative
**Patient 17**	Negative	Negative	Negative

Of these, 7 were sputum samples, and three were nasopharyngeal samples. All the oropharyngeal samples tested negative. Two patients had their sputum and nasopharyngeal samples testing positive for SARS-CoV-2 after 21 days (Table 2).

Three specimen types, oropharyngeal, nasopharyngeal and sputum, were collected from the patients 21 days after testing positive for SARS-CoV-2. Nasopharyngeal and sputum samples tested positive, while all oropharyngeal samples tested Negative.

## Discussion

This study estimated the time to SARS-CoV-2 clearance after the first RT-PCR positive result. Here, data from 480 patients confirmed as SARS CoV-2 positive and submitted at least two follow-up samples were assessed. A total of 419, representing 87%, had two consecutive negative test results. Sixty-one (13%) patients had not tested negative at the time of this study.

Literature has demonstrated that the viral shedding of infectious pathogens is significantly associated with the infectivity and transmissibility of the pathogens. Here, the median days to test PCR negative was 20 days (IQR 5 -56) (Table 1). While some individuals cleared the virus in five days, it took others as long as 56 days to achieve virus clearance. Thus, it may be important to consider individual differences in managing SARS-CoV-2 infections. The finding of a median of 20 days for virus clearance is at variance with studies that guided the updated WHO guidelines for isolation of infected SARS-CoV-2 patients,^12^, ^13^ but concordant with that of Zhou *et al.*, 2020 who reported a similar median range in Wuhan patients.^15^ Several factors including the age of the patients and type of respiratory sample collected could have contributed to the duration for testing positive. Though carried out as an exploratory study, it was observed that sputum and nasopharyngeal samples were more likely to test positive for SARS-CoV-2 after 21 days of infection compared to oropharyngeal samples. These findings were similar to those reported by Wang et al., 2020 who showed that over 70% of oropharyngeal swabs were likely to give negative results compared to nasopharyngeal swabs.^16^ This implies that sputum may be a better respiratory sample for detecting SARS-CoV-2 during the later stages of infection.

Implementing the WHO's recommendations of no retesting curbed the logistical constraints faced by countries in retesting positive cases until they obtained consecutive negative results and reduced the stigma of prolonged isolation.^17^-^19^ However, moving infected persons from isolation without confirming virus clearance could have contributed to the community spread of SARS-CoV-2 in Ghana.

This study was limited by the inability to accurately estimate the day of infection. Thus, the initial positive test date was used as a reference for analyses. This may affect the estimated duration for virus clearance. The study is further limited by our inability to culture the virus from patients to determine infectivity before the RT-PCR negative test; thus, results are based on viral RNA detection by RT-PCR, which cannot distinguish between infectious and non-infectious viruses. Also, based on the findings from the 17 patients with the three sample types, it would have been better to use only sputum samples to study viral persistence or clearance in the 480 patients since using other sample types could mask the findings. However, the data is one of the few African studies documenting the time to SARS-CoV-2 RNA clearance in infected patients who submitted follow-up samples after their initial positive test.

## Conclusion

This study showed that the median duration of SARS-CoV-2 clearance from infected persons in Ghana, as measured by two consecutive negative RT-PCR results, was 20 days (IQR: 5-56 days), irrespective of age and sex. This duration is six days longer than the 14 days accepted by WHO as the time for virus clearance, and upon which the updated guidelines for isolation were based. The findings suggest that SARS-CoV-2-infected persons in Ghana take longer to clear the virus. The finding calls for further investigations into whether patients who remain PCR positive continue to be infectious, for how long and at what virus levels to inform isolation practices in Ghana. Sputum and nasopharyngeal swabs proved more sensitive than oropharyngeal swabs to detect viral RNA as the infection progressed.
